# Addition of Propranolol in Resistant Arterial hypertension Treatment (APROPRIATE study): study protocol for a randomized double-blind placebo-controlled trial

**DOI:** 10.1186/s13063-017-1863-1

**Published:** 2017-03-14

**Authors:** G. R. Constantine, P. Ranasinghe, P. Weeratunga, C. Weeraratne, P. Galappatthy, S. Rajapakse, U. Senarath, P. Katulanda

**Affiliations:** 1 Department of Clinical Medicine, Faculty of Medicine, University of Colombo, Colombo, Sri Lanka; 2 Department of Pharmacology, Faculty of Medicine, University of Colombo, Colombo, Sri Lanka; 3 Department of Community Medicine, Faculty of Medicine, University of Colombo, Colombo, Sri Lanka

**Keywords:** Propranolol, Resistant hypertension, Randomized controlled trial, Sri Lanka

## Abstract

**Background:**

Resistant hypertension is defined as an uncontrolled blood pressure despite treatment at best-tolerated doses with at least three antihypertensive agents including a diuretic. It is an emerging public health problem. At present clinical trial data on management of resistant hypertension is limited. Management is largely based on observational studies and expert opinions. Propranolol is a nonselective beta blocker. Several studies have confirmed that propranolol has a significant hypotensive action, both when used alone and as an adjuvant therapy. At present there are no prospective, randomized, clinical studies evaluating the effectiveness of propranolol in patients with resistant hypertension. Therefore, we have designed a prospective randomized trial to evaluate the safety and efficacy of propranolol in patients with resistant hypertension.

**Methods/design:**

The study will be conducted as a randomized, double-blind, placebo-controlled clinical trial for a period of 3 months. The study has been approved by the Ethics Review Committee of the Faculty of Medicine, University of Colombo. A total of 200 adults with resistant hypertension will be recruited for the study. They will be randomly assigned to the test and placebo groups on a 1:1 ratio. The test group will receive propranolol 40 mg three times a day and the control group will receive an identical placebo capsule. The study drugs will be double blinded to both investigators and subjects. The visits and the evaluations will be done as follows: screening (visit 0), 1 month (visit 1), 2 months (visit 2) and 3 months (visit 3). The primary outcomes of the study is to find a statistically significant difference between the fall in mean systolic and mean diastolic blood pressure measured by ABPM (ambulatory blood pressure monitoring) from baseline between the two groups. Data will be analyzed using SPSS v16.

**Discussion:**

To our knowledge this is one of the first randomized controlled trials evaluating the effects of propranolol in resistant hypertension. This study will provide the necessary groundwork for future large-scale, multicentered clinical trials. The result, positive or negative, should provide a step change in the evidence guiding current and future policies regarding treatment of resistant hypertension.

**Trial registration:**

Sri Lanka Clinical Trials Registry, identifier: SLCTR/2016/002. Registered on 27 January 2016; Study protocol version 2.1.

**Electronic supplementary material:**

The online version of this article (doi:10.1186/s13063-017-1863-1) contains supplementary material, which is available to authorized users.

## Background

Resistant hypertension is defined as an uncontrolled blood pressure despite treatment (>140/90 mmHg or >150/90 mmHg in patients over the age of 60 years) with at least three antihypertensive agents at best-tolerated doses, including a diuretic [1, 2]. It is an emerging public health problem, associated with substantial morbidity and mortality [3, 4]. Studies indicate that the prevalence of resistant hypertension is 10–20% in the general hypertensive population [5, 6].

At present clinical trial data on management of resistant hypertension is limited. Management is largely based on observational studies and expert opinions. Recent studies support the use of spironolactone as the “first-line” fourth drug for treating resistant hypertension [7, 8]. However, to date there have been no randomized clinical trials comparing the effectiveness of various drug combinations in the treatment of resistant hypertension [9]. Though sympathetic denervation showed some promise in the treatment of resistant hypertension from the initial studies, subsequent studies failed to show a sustained long-term reduction of blood pressure [10].

Propranolol is a nonselective beta blocker. Several studies have confirmed that propranolol has a significant hypotensive action, both when used alone and as an adjuvant therapy [11]. However, the exact mechanism of the antihypertensive effect of propranolol is still poorly understood. It is possible that the hypotensive effect of propranolol is not only due to its peripheral beta-receptor blocking action [12]. Propranolol, with its high lipophilic tendency, achieves high concentrations in cerebrospinal fluid and blocks the central cardiac sympathetic outflow. Increased sympathetic nervous system activity has been documented in systole-diastolic hypertension and in isolated systolic hypertension [13]. Furthermore, sympathetic nervous system activity increases progressively and in parallel with stages of hypertension [14]. Central sympathetic outflow blockage may be important in reducing transient rises in blood pressure which occurs in response to various stimuli. In addition, propranolol is also known to act through the renin-angiotensin system to produce hypotension [15]. The role of stress as a risk factor for resistant hypertension also needs to be considered as drugs alone have not been very successful in controlling blood pressure in patients with resistant hypertension [16]. Propranolol is sometimes used as an anxiolytic in the premedication of surgical patients [17]. Hence, propranolol may be beneficial in reducing stress and anxiety in patients with resistant hypertension. At present there are no prospective, randomized, clinical studies evaluating the effectiveness of propranolol in patients with resistant hypertension. Therefore, we designed a prospective randomized trial to evaluate the efficacy and safety of propranolol in patients with this condition.

## Methods/design

### Objectives and hypothesis

Hypothesis: blood pressure in patients with resistant hypertension treated with propranolol will be lower than that of the control group.

Primary objective: the study aims to compare the safety and efficacy of propranolol in patients with resistant hypertension when added to a multidrug combination (at least three drugs) consisting of a diuretic and an angiotensin-converting enzyme inhibitor (ACEI)/angiotensin-II receptor blocker (ARB), a centrally acting drug, and an alpha blocker or calcium channel blocker.

Secondary objectives: (1) to describe the sociodemographic and clinical characteristics of patients with resistant hypertension at baseline, (2) to describe the complications and cardiovascular risk associations of patients with resistant hypertension at baseline, (c) to describe the impact of propranolol administration on serum/urinary electrolytes and oxidative stress in patients with resistant hypertension and (4) to assess sodium excretion in patients with resistant hypertension at baseline and following propranolol therapy.

### Study design

**Fig. 1:**
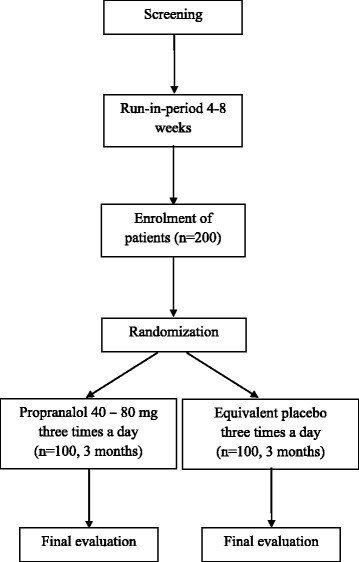
Study flow chart

**Fig. 2:**
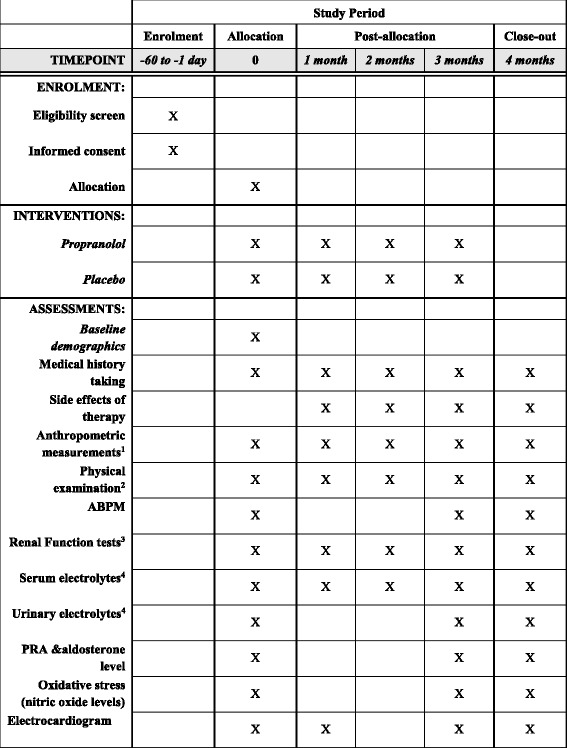
Schedule of enrollment, interventions and assessments (Standard Protocol Items: Recommendations for Interventional trials (SPIRIT) figure)

The study will be conducted as a randomized, double-blind, placebo-controlled clinical trial for a period of 3 months to assess the efficacy of using propranolol in the management of patients with resistant hypertension at the medical outpatient clinics of the National Hospital of Sri Lanka, Colombo, Sri Lanka. Figure 1 provides an overview of the study. This protocol was written following the Standard Protocol Items: Recommendations for Interventional trials (SPIRIT) Checklist (see Additional file 1). The schedule of trial enrollment, interventions and assessments is presented in Fig. 2.

### Sample size

The sample size calculation was based on formulae for analysis between differences of means. The value for alpha was set at 0.05 and the value for 1 − beta was set at 0.9. Sample sizes were calculated for a difference of systolic blood pressure by 10 mmHg and diastolic blood pressure by 5 mmHg. Standard deviation values were taken as 18 mmHg for systolic blood pressure and 10.7 mmHg for diastolic blood pressure as per prior investigations on patients with resistant hypertension. Using the above parameters the calculated sample sizes were 140 and 180 patients to detect differences in systolic and diastolic blood pressures, respectively. Using the greater number and adjusting for dropout and noncompliance rates a sample of 200 patients will be recruited with a 1:1 allocation per group.

### Population

Resistant hypertension is defined as casual blood pressure during clinical examination of more than 140/90 mmHg (in patients below 60 years of age or with diabetes mellitus) or more than 150/90 mmHg (in patients above 60 years of age) despite treatment with a multidrug combination consisting of a diuretic and an ACEI/ARB, a centrally acting drug, and an alpha blocker or calcium channel blocker [18]. All reference blood pressures will be taken as a mean of the 2nd and 3rd measurements during a single examination measured at least 3 min apart.

### Inclusion and exclusion criteria

#### Inclusion criteria

Both genders aged between 18 and 75 years and eligible for the study through screening confirming the presence of resistant hypertension as defined above.

#### Exclusion criteria

- Patients with a systolic blood pressure value over 220 mmHg requiring immediate adjustments of therapy
- Patients with moderate to severe renal insufficiency (acute or chronic) with glomerular filtration rate of less than 30 ml/min
- Patients with active bronchospastic disorders
- Heart failure classes III and IV
- Severe bradycardia (heart rate below 50/min), 2nd and 3rd degree AV block
- Pregnant or lactating women
- Patients with a history of hypersensitivity to any of the drugs under study
- Patients already using beta blockers

#### Suspension criteria

- Subject’s demand to discontinue the study
- Serious adverse events or unusual changes in clinical test results
- Principal investigator’s decision to terminate the study (low rates of compliance, complications, or unable to sustain the study for various reasons)

### Randomization

Randomization will be performed using computer-generated random number allocation tables generated by SPSS v16.0 software package (SPSS Inc., Chicago, IL, USA), based on a 1:1 allocation between groups. Patients will be assigned a recruitment number once they provide informed written consent. Each recruitment number will be subsequently allocated a computer-generated study number with a corresponding treatment kit number. These kit numbers will determine allocation to the treatment group or placebo group. The personnel administering the study medication will be blinded to the treatment group. Random number allocation tables and lists will be maintained in password-protected files accessible only in the event where unblinding is deemed necessary.

### Blinding

The study drugs are double blinded to both investigators and subjects. The drug manufacturing will be done by Astron Lanka (PVT) Ltd., Colombo, Sri Lanka, and they will be responsible for the labeling of the capsules with code numbers.

### Interventions

The treatment drug is a capsule containing propranolol 40 mg as the active ingredient. The placebo will be manufactured to have a similar appearance, shape, weight, taste and color as the propranolol 40-mg capsule. The subjects will receive either propranolol 40 mg or an identical placebo taken three times a day for a period of 3 months.

### Study groups

- Treatment group: propranolol capsule and standard treatments
- Control group: placebo capsule and standard treatments

### Study period

The study will be conducted for a period of 3 months. The visits and the evaluations will be done as follows: screening (visit 0), 1 month (visit 1), 2 months (visit 2) and 3 months (visit 3).

### Outcomes

Primary outcomes: the primary outcomes of the study are to find a statistically significant difference between the fall in mean systolic and mean diastolic blood pressure measured by ABPM (ambulatory blood pressure monitoring) from baseline between the two groups.

Secondary outcomes:

- To determine the difference in cardiovascular outcomes, including acute coronary syndrome, acute stroke and acute heart failure, between groups
- To determine the variations in plasma and urinary electrolytes between the two groups at measured endpoints
- To assess renal outcomes as measured by the glomerular filtration rate between groups
- To determine variations in plasma renin activity (PRA) and angiotensin:aldosterone ratios between the study groups
- To determine differences in oxidative stress between the study groups using nitric oxide as a surrogate marker
- To assess safety outcomes, including the incidence of serious adverse effects, between the two groups at measured endpoints

### Procedures

#### Recruitment

Participants will be recruited from the outpatient medical clinics of the National Hospital of Sri Lanka, Colombo, Sri Lanka.

#### Study schedule

The detailed items that will be measured at every visit are described in Fig. 2. During the screening visit, the following baseline examinations will be carried out. Pulse rate and blood pressure measurements in the office (a mean of the 2nd and 3rd measurements on a seated patient during a single examination, three blood pressure measurements will be taken at least 3 min apart from each other). During this screening visit patients will be evaluated for compliance and other factors for lack of control such as failure to adhere to lifestyle advices, poor blood pressure measurement technique and the use of medications that interfere with blood pressure. This will be followed by a run-in period of 4 to 8 weeks where patients identified as having resistant hypertension will be subjected to optimization of their existing drug regimen. The drug dosages will be adjusted to the maximum recommended dose or the maximum tolerated dose. After the run-in period patients will be enrolled into the study if they fulfill the inclusion and exclusion criteria. This will be the enrollment visit. In this visit serum urea, creatinine, sodium, potassium and PRA and aldosterone levels will be measured. Estimation of sodium and potassium excretion will also take place with a 24-h estimation of urinary electrolytes. Baseline oxidative stress will be assessed by estimation of nitric oxide levels. An electrocardiogram will be taken to assess cardiac rhythm. During this visit 24-h ABPM, using a monitor validated to the standards of the British Hypertension Society (BHS), will be undertaken. The mean daytime blood pressure will be calculated from values measured between 9:00 a.m. and 11:00 p.m., the mean night blood pressure from values measured between 1:00 a.m. and 6:00 a.m. The mean 24-h blood pressure will be calculated as the mean of all the measured values [19]. This will enable us to exclude patients with white coat hypertension.

After randomization patients will enter the clinical trial and will be given either propranolol 40 mg three times a day or a placebo which will be added to the patients’ routine medication. At the first two follow-up visits (visits 1 and 2) the following examinations and investigations will be performed: pulse rate and blood pressure measurements (as detailed above), measurements of serum sodium, potassium, urea and creatinine levels and an electrocardiogram. During the follow-up visits, if blood pressure is not controlled both drug and placebo dose will be doubled once (maximum dose of propranolol used in this study will be 80 mg three times daily). Three months after initiation of the drug, final examination of the patients will be carried out (visit 3), as described in Fig. 2.

### Measurement tools

#### Anthropometric measurements

Body weight will be measured using a calibrated electronic floor scale (SECA 815 by SECA GmbH & Co. Kg., Hamburg, Germany) to the nearest 0.1 kg. Height will be measured to the nearest 0.1 cm using an upright plastic portable Stadiometer (SECA 217 by SECA GmbH & Co. Kg., Hamburg, Germany). Body Mass Index (BMI) will be calculated as weight (in kilograms) divided by the square of height (in meters). Waist circumference (WC) will be measured with a nonelastic tape (SECA 203 by SECA GmbH & Co. Kg., Hamburg, Germany) at a point midway between the lower border of the rib cage and the iliac crest at the end of normal expiration. Similarly, hip circumference also will be measured at the widest part of the buttocks at the intertrochanteric level to the nearest 0.1 cm. All anthropometric measurements will be made by using standard equipment and following World Health Organization (WHO) guidelines.

#### Compliance calculation

Subjects will be asked to return any remaining drugs and their compliance will be evaluated by using the formula given below:

$$ C o m p l i a n c e\kern0.2em \left(\%\right)=\left[\frac{\left( Distributed\kern0.2em  drugs{\textstyle \mathit{\hbox{-}}} remaining\kern0.2em  drugs\right)}{Distributed\kern0.2em  drugs}\right]\times 100 $$

### Statistical analysis

Parametric and nonparametric statistical tests will be applied using the SPSS version 16 (SPSS Inc., Chicago, IL, USA) and Stata/SE 10.0 (StataCorp Inc., College Station, TX, USA) for data analysis. Descriptive data will be analyzed for linearity and will be expressed as percentages, median and mean. The two groups will be compared for uniformity on sociodemographic variables, clinical manifestations and laboratory parameters at baseline. After completion, primary and secondary outcomes will be compared in the groups using statistical methods for comparison of means and using regression analysis. For each of the outcomes, multilevel regression analysis will be used to examine differences between trial arms. For binary outcomes the model will be logistic and for continuous outcomes the model will be linear regression. All analyses will follow intention-to-treat principles and a prespecified analysis plan. Where appropriate, sensitivity analyses will be conducted (for example, control for additional covariates; and bootstrapped *p* values for skewed outcomes). In the case of missing data values, we will apply mean imputation and regression imputation where rates are low, and consider multiple imputations where they exceed 10%. A *p* value < 0.05 will be considered significant.

### Adverse effect evaluation

Described adverse effects of propranolol include: aggravation of congestive heart failure, bradycardia, hypotension, arthropathy, Raynaud’s phenomenon, hyper/hypoglycemia, depression, fatigue, insomnia, paresthesia, psychotic disorder, pruritus, nausea, vomiting, hyperlipidemia, hyperkalemia, muscle cramps, bronchospasm, dyspnea, pulmonary edema and respiratory distress. In the event of a probable adverse reaction, the following precautions would ensure timely identification and management of patients:

- Reporting: mechanisms would be put in place to ensure direct reporting of probable adverse events to the investigator by patients (via telephone, which will be available 24 h on all days)
- During follow-up visits, probable adverse events will be noted by history and examination and investigated upon in detail. The attending physician will assess for any symptoms and signs of the potential side effects. All adverse effects observed will be documented in the Case Record Form (CRF)
- All serious adverse events will be reported to the Ethics Review Committee (ERC), Faculty of Medicine, University of Colombo, and the National Pharmaco-vigilance Unit of the Department of Pharmacology, Faculty of Medicine, University of Colombo
- An independent Data Safety Monitoring Board (DSMB), consisting of clinical pharmacologists, physicians and members of the ERC, will evaluate all adverse events at regular intervals
- Termination of study: in the event of major adverse effects occurring in a significant proportion of the study population the study would be terminated pending further investigation

### Data collection

Data collection will be performed according to standard operating procedures by medically trained research assistants (RAs).

### Data and biological sample handling

Data will be entered by a minimum number of dedicated staff and saved in a dedicated password-protected computer. The data collection forms will be preserved securely. Only the investigators will have access to the computer database and the data collection forms. Blood samples will be stored in a secure facility, with secure measures taken to ensure that specimens are kept in appropriate conditions at all times when in storage. Storage technologies, with the capability of monitoring the temperature of samples around the clock, would be utilized. After each analysis has been completed and with the approval of the principal investigator, the samples stored in the storage facility may be disposed of by the sample custodian. A Sample Disposal Sheet (SDS) will be completed and kept for future reference.

### Dissemination of study finding

The results of the above study will be published in local and international peer-reviewed journals and presented at international conferences and clinical meetings.

### Ethical considerations

The study has been approved by the Ethics Review Committee (ERC) of the Faculty of Medicine, University of Colombo (EC/15/152). The trial is also registered at the Sri Lanka Clinical Trials Registry (SLCTR/2016/002). The study will be conducted in compliance with the Declaration of Helsinki and the Good Clinical Practice (GCP) guidelines. No personal details will be collected and confidentiality will be observed during the data collection process. An investigator will meet the patients in accordance with the inclusion criteria and describe the study to them. Information will be given on the purpose of the study, voluntary participation, interventions, risks hazards and benefits, confidentiality and termination of participation. The information given above will be further reinforced using an Information Sheet which has been prepared in all three languages. Following this informed written consent will be obtained using a Consent Form after clarification of any questions the participant may have regarding the study. If the participants require further clarification regarding the study the contact numbers of the investigators will be provided in the Information Sheet and the Consent Form. In the event of the change in study protocol during the course of the study, reconsent would be obtained from study participants after explaining the necessity for such change and the probable impact, if any, on the participant. The change in study protocol will be informed to all relevant parties, including the ERC.

## Discussion

In this paper, we present a clinical trial design to evaluate the effects of propranolol in those with resistant hypertension. To our knowledge this is one of the first randomized controlled trials evaluating the effects of propranolol in resistant hypertension. This study will provide the necessary groundwork for future large-scale, multicentered clinical trials. Given the current enthusiasm for using various drugs to improve blood pressure control and metabolic parameters in those with resistant hypertension, properly designed scientific evaluations are a timely requirement. However, presently there are no well-designed randomized control trials to support/refute these arguments. The result, positive or negative, should provide a step change in the evidence guiding current and future policies regarding treatment of resistant hypertension.

### Trial status

Enrollment for the trial has not yet started.

## Additional file

Additional file 1: SPIRIT 2013 Checklist: recommended items to address in a clinical trial protocol and related documents. (DOC 122 kb)

## Abbreviations

ABPM: Ambulatory blood pressure monitoring
ACEI: Angiotensin-converting enzyme inhibitor
ARB: Angiotensin-II receptor blocker
BHS: British Hypertension Society
BMI: Body Mass Index
CRF: Case Record Form
DSMB: Data Safety Monitoring Board
ERC: Ethics Review Committee
GCP: Good Clinical Practice
PRA: Plasma renin activity
SDS: Sample Disposal Sheet
SLCTR: Sri Lanka Clinical Trials Registry
WC: Waist circumference
WHO: World Health Organization
